# Prevalence, type, and prognosis of ocular lesions in shelter and owned-client dogs naturally infected by *Leishmania infantum*

**DOI:** 10.14202/vetworld.2016.633-637

**Published:** 2016-06-22

**Authors:** Simona Di Pietro, Valentina Rita Francesca Bosco, Chiara Crinò, Francesco Francaviglia, Elisabetta Giudice

**Affiliations:** 1 Department of Veterinary Science, University of Messina, Polo Universitario Annunziata, 98168 Messina, Italy; 2 DVM, Veterinary Medical Centre S. Chiara, Viale Vittorio Veneto, 96014 Floridia (SR), Italy; 3 DVM, Local Public Health Unit (ASP) of Palermo, Via G. Cusmano 24, 90141, Palermo, Italy; 4 Department of Chemical, Biological, Pharmaceutical and Environmental Sciences, University of Messina, Viale F. Stagno d’Alcontres 31, 98168 Messina, Italy

**Keywords:** dog, follow-up, leishmaniasis, ocular lesions, post-treatment uveitis

## Abstract

**Aim:**

The point prevalence of ocular lesions due to leishmaniasis was evaluated in 127 dogs living in a municipal shelter placed in a highly endemic area (Sicily, Italy). Moreover, the period prevalence, the type, and prognosis of lesions due to leishmaniasis were evaluated in 132 dogs with ocular pathologies referred to a Veterinary Teaching Hospital (VTH) in the same endemic area over a 3-year period.

**Materials and Methods:**

All the dogs were submitted to ophthalmological examination. The diagnosis of leishmaniasis was made by cytological, serological (immune-fluorescent antibody test), and molecular (quantitative polymerase chain reaction) tests.

**Results:**

The point prevalence of ocular lesions in 45 shelter dogs with leishmaniasis was 71.11% (45/127 dogs). The most frequent ocular lesion was blepharitis (50%) while anterior uveitis was observed in only 9.37% of cases. The period prevalence of ocular lesions due to leishmaniasis in the VTH group was 36.36% (48/132 dogs). In both groups, most of the lesions were bilateral and involved the anterior segment. Anterior uveitis was the most frequent ophthalmic finding in client-owned dogs (37.50%), but it occurred in only 9.37% of the shelter dogs. Keratouveitis often occurred during or after antiprotozoal treatment (14.58%; 7/48). In this study, the healing of eye injury following systemic antiprotozoal treatment was recorded in about half of cases (48%; 12/25 dogs), in which follow-up was possible. In more than 1/3 of cases (36%; 9/25), there was an improvement, but it was necessary to associate a long-term topical treatment; most of them, as well as those who had not responded to systemic therapy (16%; 4/25), had anterior uveitis or keratoconjunctivitis sicca.

**Conclusions:**

Ocular manifestations involve up to 2/3 of animals affected by canine leishmaniasis and lesions account for over 1/3 of ophthalmic pathologies observed at a referral clinic in an endemic area. The occurrence of anterior uveitis is more frequent in client-owned dogs than in shelter dogs. The onset of keratouveitis during or after antiprotozoal treatment could be attributed to the treatment or to a recurrence of the systemic form. The post-treatment uveal immune reaction, already observed in humans, could explain the difference in the frequency of keratouveitis between client-owned and shelter dogs, which have never been treated.

## Introduction

Many endo- or ecto-parasites may affect animals and their adverse effects on health, production, and welfare have been repeatedly documented [1,2]. Furthermore, many parasitic diseases are zoonoses and thus a severe public health concern [3,4].

Canine leishmaniasis is a chronic and severe systemic disease caused by the protozoan parasite *Leishmania infantum*. It is endemic in the Mediterranean area, and the infection vectors are sand flies (*Phlebotomus* spp.).

Clinical signs associated with leishmaniasis are highly variable as the consequence of numerous different pathogenic mechanisms and different immune responses of individual hosts [5]. Ocular signs occur in 16-80% of affected dogs [5-7]. Blepharitis, keratoconjunctivitis, and anterior uveitis were described as the most frequent signs [6]. Adnexal lesions such as periocular alopecia, eyelid nodule, and keratoconjunctivitis sicca (KCS) are also common. Previous clinical studies have reported the occurrence of KCS in dogs with leishmaniasis varying from 2.8% to 26.43% [6-8]. Other reported ocular manifestations include cyclitis, chorioretinitis, retinal detachment, cataract, glaucoma, and orbital cellulitis [5].

In literature, there is a considerable variability in the prevalence of eye lesions observed during canine leishmaniasis, likely due to differences in the canine population and the clinical approach (generic or specialist) evaluated. Although studies conducted on both shelter and client-owned dogs are numerous, there is a lack of knowledge in the comparison between these types of population.

The aim of this study was to evaluate the point prevalence of ocular lesions in shelter dogs and the period prevalence, type, and prognosis of ocular lesions associated to leishmaniasis in dogs referred to a specialty clinic both from the same endemic area.

## Materials and Methods

### Ethical approval

All treatments, housing, and animal care reported in this study were reviewed and approved in accordance with the standards recommended by the Guide for the Care and Use of the Laboratory Animals and the EU Directive 2010/63/EU for animal experiments. The pet owners consented to have their animals involved in this study.

### Canine population

A total of 259 dogs, of various breeds, sex, and age (Table-1), were enrolled in the study: 127 dogs living in a municipal shelter in Palermo (Northern Sicily, Italy) and visited in September 2007; 132 dogs referred to the ophthalmology unit of the Veterinary Teaching Hospital (VTH) of the Department of Veterinary Sciences of the University of Messina (Northern Sicily) over a 3-year period (2004-2007). All animals were submitted to general physical examination and ophthalmological assessment.

**Table-1:** Signalment of canine population.

Group	Dogs	Sex		Age (years)		
	
		Male	Female	<1	1-4	>4
Shelter group	127	53	74	25	42	60
VTD group	132	86	46	12	28	92
Total	259	139	12	37	70	152

VTH=Veterinary teaching hospital

### Canine samples

Popliteal lymph node aspirates were obtained from each dog using a thin biopsy needle. A thin smear was performed immediately after collection. When occurred, smears were performed on nodular and/or ulcerative lesions. Smears were stained with May-Grünwald Giemsa and examined under an optical microscope to determine whether amastigote forms of *L. infantum* were present. Each smear was examined for 10 min (100 microscopic fields) under a 100× oil immersion objective lens.

On each dog, peripheral blood was obtained from the jugular vein and equally distributed into tubes with and without ethylenediaminetetraacetic acid, for biomolecular testing (quantitative polymerase chain reaction [qPCR]) and serologic (immune-fluorescent antibody test [IFAT]), respectively. For the former tests, samples were also collected from left popliteal lymph node and conjunctival swabs (exfoliative epithelial cells collected using sterile cotton swabs rubbed robustly back and forth once in the lower conjunctival sac).

All serological and molecular tests were performed at the Italian National Reference Centre for Leishmaniasis (CReNaL) of the Istituto Zooprofilattico Sperimentale della Sicilia, Palermo, Italy.

The IPT1 ZMON1 *L. infantum* promastigotes were used as antigen for IFAT assay and to construct the standard curve for qPCR. The IPT1, taken from the collection of CReNaL, were grown in Tobie agar medium Evans modified [9,10].

In the IFAT assay, the antigen was fixed on multispot microscope slides (Bio-Merieux, Marcy L’Etoile, France) in acetone bath. The dog sera were serially diluted (1:40-1:5120) in pH 7.2 phosphate-buffered saline (PBS) and added to the antigen-coated wells. The slides were incubated for 30 min at 37°C. Positive and negative controls were included in each series of analyzed samples. Fluorescent staining was performed using an anti-dog immunoglobulin G labeled with fluorescein isothiocyanate (Sigma-Aldrich, Saint Louis, MO, USA) diluted 1:200 in PBS. The slides were examined using a fluorescence microscope (Leica DM 4000B, Heerbrugg, Switzerland). The IFAT results were regarded as positive when dilutions of the sera gave an evident yellowish-green fluorescent signal on microscopic observation while non-reactive samples showed no color. The cutoff value was established at a serum dilution of 1:40. The positive control consisted in a known title serum of a dog with positive cultural isolation. The negative control consisted in serum from a dog which was negative to cultural test.

In tissue samples, the DNA was extracted using EZNA. Tissue DNA Kit (Omega Biotech VWR) according to the manufacturer’s instructions. The PCR test was targeted on a 123 bp fragment inner the constant region in the minicircle kinetoplast DNA (NCBI accession number AF291093) and was carried out as previously described [11]. The primers and probe were chosen with the assistance of Primer Express Software (Applied Biosystems). The primer sequences were: QLK2-U 5’-GGCGTTCTGCGAAAACCG-3’; QLK2-D5’-AAAATGGCATTTTCGGGCC-3’; while the associated probe was: 5’-TGGGTGCAGAAATCCCGTTCA-3’ 5’FAM and 3’ BHQ labeled. Each amplification was performed in duplicate. The thermal cycling conditions comprised an initial incubation for 2’ at 50°C for uracil-N-glycosylase activity. This step was followed by a 10’ denaturation at 95°C and 45 cycles at 95°C for 15” and 60°C for 1’ each. The quantity of DNA in the samples examined was detected by comparison with a standard curve. The DNA concentration was estimated by spectrophotometric determination of A260 and A280 and by gel electrophoresis. On the basis of the linearity in the fluorescent signal through the serial standard DNA dilutions, PCR test and dedicated software (SDS Applied Biosystems) permitted detection of parasitic charge lower than 1 cell/mL of the tissue matrices. Two replicates of six different concentrations of *L. infantum* DNA were tested in the same run. In this way, we performed intra- and inter-assay comparison of the obtained signal for each DNA concentration.

## Results

### Shelter group

**Figure-1:**
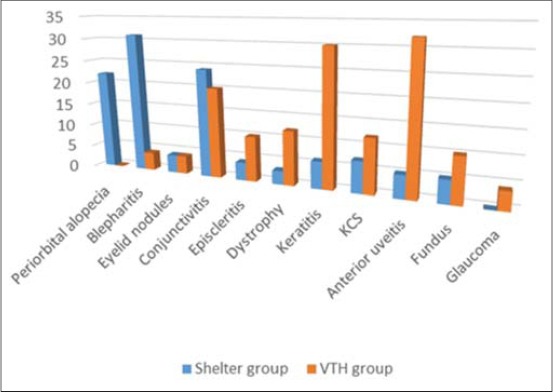
Type and frequency of ocular lesions in 80 leishmaniotic dogs (shelter group and Veterinary Teaching Hospital group).

Out of the 127 examined dogs, 45 (35.43%) were affected by leishmaniasis. The diagnosis was made on the basis of the positivity of serology (IFAT, ≥1:640), microscopy and at least lymph node qPCR. 32 of these animals (point prevalence: 71.11%) showed ocular and periocular lesions referable to the disease. Ocular lesions were bilateral in 27 dogs (84.37%), 20 of which had more than one ocular sign. In all animals and eyes (100%), the lesions involved the anterior segment and in one dog the lesion involved the anterior segment in one eye and both segments in the other eye. The type and frequency of ocular lesions in 32 shelter dogs with leishmaniasis (59 affected eyes) are reported in Table-2 and Figure-1. The most frequent ocular lesion was blepharitis (16/32, 50%); anterior uveitis was observed in only 3 dogs (9.37%).

**Table-2:** Type and frequency of ocular lesions in 48 client-owned dogs (VTH group) and in 32 shelter dogs (shelter group) with leishmaniasis.

Ocular lesion	VTD group		Shelter group	
	
	Dogs (%)	Eyes (%)	Dogs (%)	Eyes (%)
Periorbital alopecia	0	0	11 (34.37)	22 (37.29)
Blepharitis	2 (4.17)	4 (4.44)	16 (50.00)	31 (52.54)
Eyelid nodules	4 (8.33)	4 (4.44)	3 (9.37)	4 (6.78)
Conjunctivitis	10 (20.83)	20 (22.22)	12 (37.50)	24 (40.68)
Episcleritis	6 (12.50)	10 (11.11)	2 (6.25)	4 (6.78)
Dystrophy	6 (12.50)	12 (13.33)	2 (6.25)	3 (5.08)
Keratitis	16 (33.33)	30 (33.33)	4 (12.50)	6 (10.17)
KCS	6 (12.50)	12 (13.33)	4 (12.50)	7 (11.86)
Anterior uveitis	18 (37.50)	32 (35.55)	3 (9.37)	5 (8.47)
Fundus	6 (12.50)	10 (11.11)	3 (9.37)	5 (8.47)
Glaucoma	2 (4.17)	4 (4.44)	-	-
Anterior district	46 (95.83)	84 (93.33)	32 (100)	59 (100)
Posterior district	8 (16.67)	14 (15.55)	3 (9.37)	5 (8.47)
Sub total	48 (100)	90 (93.75)	32 (100)	59 (92.19)
No lesion	-	6 (6.25)	-	5 (7.81)
Total	48 (100)	96 (100)	32 (100)	64 (100)

VTD=Veterinary teaching hospital, KCS=Keratoconjunctivitis sicca

### VTH group

Out of the 132 dogs with ocular pathologies referred to the ophthalmology unit of the VTH, 58 (43.94%) were affected by leishmaniasis, resulting positive to the diagnostic tests. 48 of these animals showed ocular and periocular lesions referable to the disease (period prevalence: 36.36%). Ocular lesions were bilateral in 42 cases (87.50%); 28 dogs (58.33%) had more than one ocular sign. The ocular lesions involved the anterior segment in 46 dogs (95.83%) and the posterior segment in 8 dogs (16.67%). In 4 dogs (8.33%), the lesions involved both segments. The distribution of the ocular lesions in 48 referred dogs with leishmaniasis (90 eyes) is reported in Table-2 and Figure-1. The most common lesions were anterior uveitis which occurred in 18 dogs (37.50%).

In 41 dogs (85.42%), the ocular lesions were recorded when leishmaniasis was diagnosed, whereas in the other 7 dogs (14.58%) the lesions (keratouveitis) occurred during or after the specific treatment; in 1 case, uveitis developed on the 15^th^ day of therapy; in 6 cases, the lesions appeared several weeks after the beginning of a previous cycle of antimonial therapy (mean 132.5±31.2 days; median: 129 days; range: 90-179 days).

### Follow-up study

About 25 cases of the VTH group were evaluated to ascertain response and prognosis of ocular lesions to antiprotozoal therapy, with N-methylglucamine antimoniate (100 mg/kg daily for 60 days, subcutaneously), combined with allopurinol (10 mg/kg twice daily for 6-12 months orally). The mean follow-up was 8.7 months (range: From 2 months to 2 years).

In 12 dogs (48%), a complete resolution of ocular lesions was obtained after antiprotozoal therapy alone (mean: 14.5±10.4 days; median: 12.5 days; range: 5-45 days). The lesions were as follows: Nodular blepharitis (n=2 cases), keratoconjunctivitis (n=2), keratitis (n=2), and keratouveitis (n=6). In 9 dogs (36%), the antiprotozoal treatment determined an improvement of ocular lesions, while healing was achieved only after a long topical therapy (14-56 days) with non-steroidal or steroidal anti-inflammatory, cyclosporine, artificial tears, midriatic/cycloplegics, and/or antiglaucoma medications, whether or not associated with systemic anti-inflammatories. The lesions were: KCS (n=3), keratouveitis (n=2), anterior uveitis (n=3), and post-uveitic glaucoma (n=1). In the other 4 dogs (16%), the combined treatment did not lead to healing but produced only a slight improvement. All the dogs had serious and inveterate lesions: Blepharoconjunctivitis associated to keratouveitis (n=1), glaucoma (n=1), and KCS (n=2) (Table-3).

**Table-3:** Response of ocular lesions to antiprotozoal treatment alone or combined with ocular therapy and healing times of in 25 leishmaniotic dogs referred to a Veterinary Teaching Hospital.

Response to treatment	Antiprotozoal therapy		Follow-up (days)			Total N (%)
	
	Alone (%)	Plus ocular therapy (%)	Mean	Median	Range	
Complete healing	12 (48)		14.5±10.4	12.5	5-45	21 (84)
		9 (36)	31.1±14.5	28.0	14-56	
No healing		4 (16)	403.7±219	365	180-705	4 (16)
Total	12 (48)	13 (52)				25 (100)

## Discussion

The present research was carried out in two different areas of Sicily (Southern Italy) with a similar high-endemicity for leishmaniasis [12].

During canine leishmaniasis, ocular manifestations involved up to 2/3 of the affected animals (shelter group), and the lesions due to leishmaniasis are more than 1/3 of the ophthalmic pathologies observed in a referral clinic in an endemic area.

Anterior uveitis was the most frequent ophthalmic finding in client-owned dogs (37.50%), but it occurred in only 9.37% of the shelter dogs. As the operators and research methodology as well as the geographical area of study were the same, the only variable that may explain the different percentages of occurrence is the type of sample, client-owned dogs being well cared for while shelter dogs are almost never treated.

The occurrence of anterior uveitis in seven animals (28%) during or after the antiprotozoal treatment could be attributed to the treatment itself or to a recurrence of the systemic form. The nodular form of uveitis, which often develops after initiation of antiprotozoal therapy, may have an allergic basis resulting from the death of the organism in tissues [6], similarly to what is observed for post-kala-azar anterior uveitis in humans [13].

The post-treatment uveal immune reaction may explain the difference in the frequency of uveitis between client-owned dogs and shelter dogs.

Periocular lesions, such as blepharitis and periocular alopecia, are more frequent in shelter dogs (50% and 34.37%, respectively) than in VTH group (33.33% and 0%, respectively). Eyelid lesions are also often considered as dermatological signs (periocular dermatitis), and they are not referred to an ophthalmologist.

**Figure-2:**
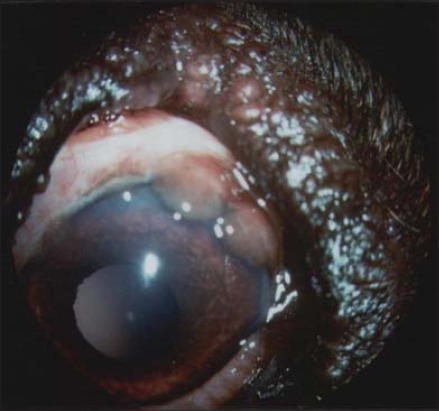
Granulomatous lesions at lateral corneal limbus due to *Leishmania* spp.

**Figure-3:**
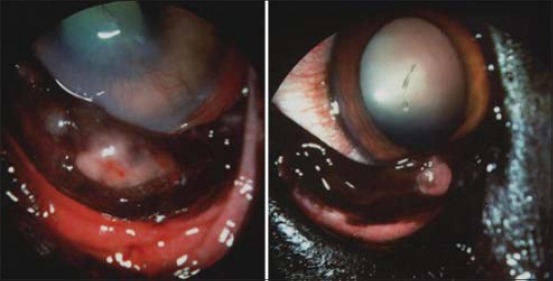
Nodular lesions on cornea, conjunctiva, and eyelids due to *Leishmania* spp.

**Figure-4:**
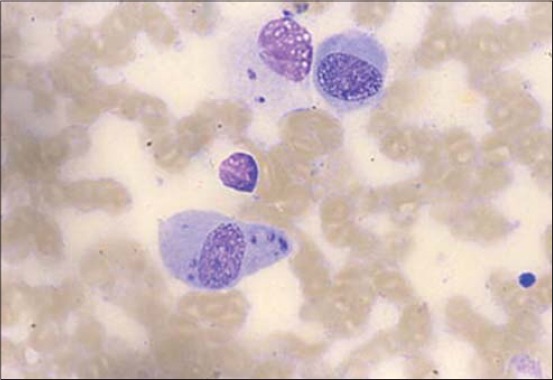
Microscopic examination of an ocular nodule that showed the presence of amastigote parasites.

Granulomatous nodular lesions were recorded in both VTH and shelter groups. In two client-owned dogs, this kind of lesion was found in lateral corneal limbus (Figure-2) and also described in previous papers [6] and similar to granulomatous episcleritis observed in Collies and other breeds [14]. Nodular lesions in cornea, conjunctiva, and eyelids were also found in five client-owned dogs and three shelter dogs (Figure-3). The microscopic examination of granulomas always showed the parasite (Figure-4). This suggests that cytology is useful in these lesions but also that inflammation of the conjunctiva and cornea could be directly related to the protozoan rather than to an altered immune response due to infection [6]. The granulomas on the eyelid margin could also be a reaction at the vector biting site, as hypothesized by other researchers [6,11].

In this study, the healing of eye injury following systemic antiprotozoal treatment was recorded in about half the cases (48%), in which follow-up was possible. In over 1/3 of cases (36%), there was an improvement, but it was necessary to associate a long-term (months) topical treatment; most of them, as well as those which had not responded to combined therapy (16%), had anterior uveitis or KCS. These findings agree with previous observations [6,15], in which a favorable prognosis for the lesions of the adnexa and for most inflammatory intraocular lesions was envisaged, except for anterior uveitis which is often refractory to antiprotozoal therapy and much more difficult to treat.

## Conclusion

Many aspects of leishmaniasis have not yet been clarified, and ocular involvement can direct toward a diagnosis of the disease, especially when it is the only clinical sign or in the case of the specific granulomatous lesions. Special attention should also be paid to uveal lesions that may be particularly severe and difficult to treat and whose appearance may be linked to causal therapy.

Given the complexity of ocular lesions and their varied response to therapy, we need an earlier and accurate diagnosis, obtained by appropriate means, the use of which may require the expertise of a specialist.

## Authors’ Contributions

SDP, VRFB, and EG designed the study. The experiment was done by SDP, VRFB, CC, FF, and EG, whereas laboratory work was done by EG and VRFB. All the authors participated in data analysis, draft, and revision of the manuscript. All authors read and approved the final manuscript.

## References

[1] Pugliese A, Di Pietro S, Giudice E (2006). Clinical and diagnostic patterns of leishmaniasis in the dog. Vet. Res. Commun.

[2] Giudice E, Domina F, Britti D, Di Pietro S, Pugliese A (2003). Clinical findings associated with *Borrelia burgdorferi* infection in the dog. Vet. Res. Commun.

[3] Giudice E, Di Pietro S, Alaimo A, Blanda V, Lelli R, Francaviglia F, Caracappa S, Torina A (2014). Molecular survey of *Rickettsia felis* in fleas from cats and dogs in Sicily (Southern Italy). PLoS One.

[4] Giudice E, Di Pietro S, Gaglio G, Di Giacomo L, Bazzano M, Mazzullo G (2013). Adult of *Dirofilaria repens* in a dog with recurrent multiple subcutaneous nodular lesions. Parasitol. Res.

[5] Peña M.T, Naranjo C, Klauss G, Fondevila D, Leiva M, Roura X, Davidson M.G, Dubielzig R.R (2008). Histopathological features of ocular leishmaniasis in the dog. J. Comp. Pathol.

[6] Peña M.T, Roura X, Davidson M.G (2000). Ocular and periocular manifestations of leishmaniasis in dogs:105 cases (1993-1998). Vet. Ophthalmol.

[7] Naranjo C, Fondevila D, Altet L, Francino O, Rios J, Roura X, Peña T (2012). Evaluation of the presence of *Leishmania spp*. by real-time PCR in the lacrimal glands of dogs with leishmaniasis. Vet. J.

[8] Ciaramella P, Oliva G, De Luna R, Ambrosio R, Cortese L, Persechino A, Gradoni L, Scalone A (1997). A retrospective clinical study of canine leishmaniasis in 150 dogs naturally infected by *Leishmania infantum*. Vet. Rec.

[9] Titus R.G, Marchand M, Boon T, Louis J.A (1985). A limiting dilution assay for quantifying *Leishmania* major in tissues of infected mice. Parasite Immunol.

[10] Tobie E.J, Von Brand T, Mehelman B (1950). Cultural and physiological observations on *Trypanosoma rhodiense* and *Trypanosoma gambiense*. J. Parasitol.

[11] Manna L, Reale S, Vitale F, Picillo E, Pavone L.M, Gravino A.E (2008). Realtime PCR assay in *Leishmania*-infected dogs treated with meglumine antimoniate and allopurinol. Vet. J.

[12] Lombardo G, Pennisi M.G, Lupo T, Chicharro C, Solano-Gallego L (2014). Papular dermatitis due to *Leishmania infantum* infection in seventeen dogs:Diagnostic features, extent of the infection and treatment outcome. Parasit. Vectors.

[13] el Hassan A.M, Khalil E.A, el Sheikh E.A, Zijlstra E.E, Osman A, Ibrahim M.E (1998). Post kala-azar ocular leishmaniasis. Trans. R. Soc. Trop. Med. Hyg.

[14] Esson D.W (2015). Clinical Atlas of Canine and Feline Ophthalmic Disease.

[15] Roze M (2004). Ocular manifestations of canine leishmaniasis. Diagnosis and treatment.

